# Tangle- and contact-free path planning for a tethered mobile robot using deep reinforcement learning

**DOI:** 10.3389/frobt.2024.1388634

**Published:** 2024-09-02

**Authors:** Ryuki Shimada, Genya Ishigami

**Affiliations:** Graduate School of Integrated Design Engineering, Faculty of Science and Technology, Keio University, Yokohama, Japan

**Keywords:** tethered mobile robot, path planning, homotopy class, reinforcement learning, deep Q-network

## Abstract

This paper presents a tangle- and contact-free path planning (TCFPP) for a mobile robot attached to a base station with a finite-length cable. This type of robot, called a tethered mobile robot, can endure long-time exploration with a continuous power supply and stable communication via its cable. However, the robot faces potential hazards that endanger its operation such as cable snagging on and cable entanglement with obstacles and the robot. To address these challenges, our approach incorporates homotopy-aware path planning into deep reinforcement learning. The proposed reward design in the learning problem penalizes the cable-obstacle and cable-robot contacts and encourages the robot to follow the homotopy-aware path toward a goal. We consider two distinct scenarios for the initial cable configuration: 1) the robot pulls the cable sequentially from the base while heading for the goal, and 2) the robot moves to the goal starting from a state where the cable has already been partially deployed. The proposed method is compared with naive approaches in terms of contact avoidance and path similarity. Simulation results revealed that the robot can successfully find a contact-minimized path under the guidance of the reference path in both scenarios.

## 1 Introduction

A tethered mobile robot can perform exploration for a long duration with a continuous power supply and stable communication through its cable. In addition, the robot can use the cable as a lifeline on steep slopes or cliffs to prevent falling or to hook it onto fixed obstacles. Their typical applications include the exploration of nuclear power plants (Nagatani et al., 2013), underwater areas, subterranean spaces (Martz et al., 2020), and lunar/planetary slopes and caves (Abad-Manterola et al., 2011; Schreiber et al., 2020). Untethered mobile robots have indeed explored various environments, such as uneven terrains or cluttered areas, but we believe that tethering mobile robots is such a powerful solution that will allow for exploration into previously uncharted territories while ensuring power, communication, and fall safety. However, difficulties arise when we try to deploy the tethered robot system. This is because the path planning algorithms for conventional mobile robots cannot be applied directly to tethered robots owing to constraints such as the cable length and the cable’s interaction with the robot and obstacles.

A major challenge in path planning for tethered mobile robots has been computing the shortest path to the target point, considering the cable length and cable-obstacle interaction. This issue has been studied extensively in Xavier (1999), Brass et al. (2015), Abad-Manterola et al. (2011), Kim et al. (2014), Kim and Likhachev (2015), and Sahin and Bhattacharya (2023). The motivation stems from the fact that the workspace of a tethered robot is theoretically a circular shape, whose center is the anchor point of the cable when there are no obstacles in the environment. However, this is not the case when obstacles are present. In early research, visibility graph-based approaches were proposed in Xavier (1999), Brass et al. (2015), and Abad-Manterola et al. (2011). These studies assumed that the cable automatically coiled to maintain its tension at all times.

Research in this area has gained momentum only since the work of homotopy-aware path planning, which was first proposed by Igarashi and Stilman (2010) and mathematically refined by Kim et al. (2014). The essence of this approach is to topologically encode a path by its placement with respect to the obstacles in the environment. The usefulness of this method can be observed in its application to exploration problems (Shapovalov and Pereira, 2020) and 3D environments (Sahin and Bhattacharya, 2023).

Recent studies have focused on avoiding cable-robot and cable-obstacle contacts. Path planning with cable-robot avoidance was developed in Yang et al. (2023), whereas cable-obstacle contact is admissible. Our previous study, Shimada and Ishigami (2023), proposed a waypoint refinement method based on the distance from the cable base, curvature, and proximity to obstacles, which was formulated using an artificial potential field. However, this method is only applicable when the cable is initially stored in a retractable mechanism and cannot be used when the cable is initially deployed in the environment.

Despite such intensive research effort, a comprehensive approach that balances the three objectives—overcoming cable length constraints, avoiding cable-robot contact, and avoiding cable-obstacle contact—has not been developed yet. To this end, we must consider the following two issues: 1) the global path generated by conventional mobile robot methods cannot be used directly as a path for a tethered robot, and 2) the robot has no knowledge of the cable dynamics and cannot directly control the cable position, because of the underactuated nature of the tethered robot system.

**FIGURE 1:**
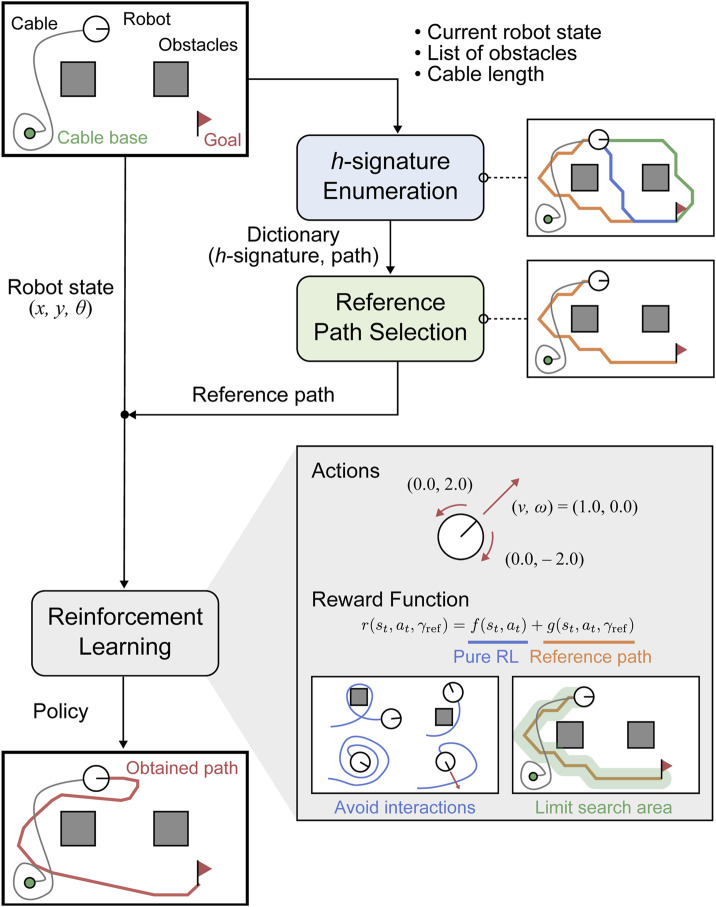
Flowchart of the proposed algorithm: Tangle- and Contact-free Path Planning (TCFPP) method. The algorithm aims to generate paths that minimize cable-obstacle and cable-robot contact.

In this study, we aim to solve a path planning problem that considers the cable length constraints and minimizes the cable-obstacle and cable-robot contacts. Our approach, a tangle- and contact-free path planning (TCFPP) algorithm uses deep reinforcement learning (DRL) with a homotopy-aware reference path guidance (Figure 1). The reward function in DRL has two components: the first guides contact avoidance and the second suppresses the deviations from the reference path. We built a customized Gymnasium environment using a kinematics-based robot model and a position-based cable model. A standard DRL algorithm, Deep Q-Network (Mnih et al., 2015) can successfully determine an effective path in a given environment. We evaluated the proposed method through simulations from two aspects: contact avoidance and similarity with the paths of naive approaches.

The following are the key contributions of this study:

• We develop a method for selecting a reference path for a tethered mobile robot from enumerated feasible paths in terms of homotopy class.
• We propose TCFPP, a path planning method for a tethered mobile robot that considers cable-obstacle and cable-robot avoidance using DRL with homotopy-aware reference path guidance.
• We show that the proposed method effectively balances path shortness with maintaining the distance from obstacles.

The remainder of this paper is organized as follows. Section 2 introduces the concepts of homotopy class of path and h -signature, and reinforcement learning with a reference path. Section 3 provides the problem statement, the two scenarios to be tested. Section 4 presents the tangle- and contact-free path planning method for a tethered mobile robot. Section 5 describes the tethered mobile robot model with a kinematics-based robot model and position-based cable model for simulations. Section 6 shows the simulation results and discusses the performance of our method. Section 7 concludes this work.

## 2 Preliminaries

The proposed method uses DRL with hommotopy-aware reference path. This section introduces the notion of homotopy class of path and the basics of reinforcement learing with a reference path.

### 2.1 Homotopy class of paths

The notion of *homotopy class of paths* plays an important role in capturing their nature based on their topological relations to obstacles in the environment, rather than their geometric properties, such as length, curvature, or smoothness. Here we outline the concept of a homotopy class and introduce h *-signature*, defined in Kim et al. (2014), which is a unique identifier of the class, and its operation.

**FIGURE 2:**
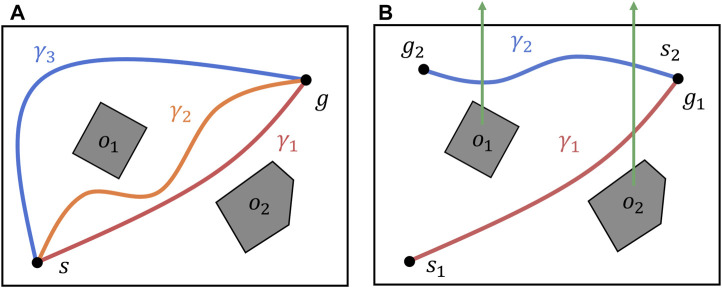
Homotopic relation between paths in an environment with two obstacles. **(A)** Three paths in an environment with two obstacles. Paths $\gamma_{1}$ and $\gamma_{2}$ belong to the same homotopy class; paths $\gamma_{1}$ and $\gamma_{3}$ belong to different homotopy classes. **(B)** Concatenation of h -signatures. The h -signature of a path is computed by checking its intersections with the green arrows starting from the center of the obstacles. Two h -signatures can be concatenated if the end point of one path corresponds to the start point of the other.

Consider two paths that share the same start and end points. The two paths belong to the same homotopy class if and only if they can be deformed into each other without intersecting any obstacles. For example, in Figure 2A, the paths $\gamma_{1}$ and $\gamma_{2}$ are in the same homotopy class, whereas $\gamma_{1}$ and $\gamma_{3}$ are not. To identify the homotopy class of the paths, a metric called the h *-signature* is used. This metric is determined by considering the intersections of a path with parallel arrows that extend from the center of the obstacles to the north of the map (green arrows in Figure 2B). The sign of the h -signature changes based on the direction in which these arrows are crossed: a left-to-right passage assigns the obstacle’s identifier, and a right-to-left passage appends a sign-reversed identifier. It should be noted that the h -signature can be an empty list if the path does not cross any arrows. The h -signature of the path is determined by checking the intersection of the path and the arrows from the starting point to the end point. Let h(γ) denote the h -signature of path γ . The h -signatures of the two paths $\gamma_{1}$ and $\gamma_{2}$ in Figure 2B, *i.e.*, $h\left(\gamma_{1}\right)$ and $h\left(\gamma_{2}\right)$ , are computed as $\left[o_{2}\right]$ and $\left[-o_{2},-o_{1}\right]$ , respectively.

In addition, the h -signatures for the two paths can only be concatenated when the end point of one coincides with the start point of the other. In this operation, the same identifiers with reversed signs are removed from the list. For paths $\gamma_{1}$ and $\gamma_{2}$ in Figure 2B, one can concatenate their list as $\left[o_{2},-o_{2},-o_{1}\right]$ ; then, this can be simplified as $\left[-o_{1}\right]$ . In the following part, we express the concatenation using a “ ♢ ” operator. This operation can be written as $h\left(\gamma_{1}\right)♢h\left(\gamma_{2}\right)=\left[o_{2},-o_{2},-o_{1}\right]=\left[-o_{1}\right]$ . Further details regarding the definition and operation of the h -signature can be found in Kim et al. (2014).

### 2.2 Reinforcement learning with reference path

We consider the standard RL problem with reference path $\gamma_{\text{ref}}$ . RL is formulated as a Markov decision process (MDP) with a four-element tuple, (S,A,T,R) , where S denotes the state space, A is the action space, T is the state transition function, and R is the reward signal. The reference path provides the agent with global insights, thus enhancing its ability to find the optimal path faster and improving local motion for effective contact avoidance. We convey the reference path information to the agent via a reward function, as presented in Ota et al. (2020). At time step *t*, the state and action are represented by s_t_ in *S* and a_t_ in *A*, respectively. Then, the reward function can be written as follows:

$$r\left(s_{t},a_{t},\gamma_{\text{ref}}\right)=f\left(s_{t},a_{t}\right)+g\left(s_{t},a_{t},\gamma_{\text{ref}}\right).$$ (1)

The first term, $f\left(s_{t},a_{t}\right)$ , represents the reward in pure RL. The second term, $g\left(s_{t},a_{t},\gamma_{\text{ref}}\right)$ , denotes the reward function related to a reference path. This function imposes penalties for deviating from the reference path while learning, and simultaneously provides positive rewards for reaching each waypoint of the reference path.

## 3 Problem statement

This study addresses a path planning problem that considers the constraints imposed by the cable length and minimizes the cable-obstacle and cable-robot contacts. The goal was to avoid entanglement and contact between the three entities: the robot, cable, and obstacles. Although these interactions do not always endanger the robot, they potentially impede its safe operation.

In a tethered mobile robot path planning, there are two primary scenarios based on the initial state of the cable: Unreeling Cable and Handling Deployed Cable. In the unreeling cable scenario, the cable and robot follow nearly identical paths, thereby focusing on avoiding cable-obstacle contact. In contrast, the handling deployed cable scenario treats the cable as an additional obstacle, thus requiring paths that avoid both cable-obstacle and cable-robot contacts.

Figure 1 illustrates the flowchart of the proposed algorithm. This algorithm inputs the robot’s current and target positions, cable placement, and cable length, and aims to output paths that minimize cable-related contact with the guidance of a homotopy-aware reference path. For the computation of the reference path, we first enumerate all paths with different homotopy classes that connect the robot’s current and target points, and then select the shortest and reachable path as the reference path, taking into account cable length constraints.

A key assumption is that the decision-making entity in DRL, which we call the agent, has no knowledge of the cable behavior, and its interaction with the environment can be obtained only via reward signals.

## 4 Proposed method: TCFPP

The proposed method comprises three main modules: 1) enumeration of shortest paths with distinct h -signatures, 2) selection of a reference path from the paths enumerated in 1), considering the constraints imposed by the cable length, and 3) training of an agent using DRL with the homotopy-aware reference path (Figure 1).

### 4.1 Step one—Enumerating h -signatures

The objective of this step is to enumerate all possible h -signatures and to find the shortest path with each h -signature. Algorithm 1 first enumerates paths in order of length—from shorter to longer—using Yen’s k -shortest path routing algorithm (lines 2–7) and then determines the h -signature of each path and keeps the shortest one among them (lines 8–19). We present below the procedural details of Algorithm 1.

The algorithm initializes two lists A and B to store paths and a dictionary D that takes the h -signatures as key and a shortest path with the h -signature as value (lines 2–3). To find the possible h -signature under given configurations of the robot and obstacles, the proposed algorithm aims to enumerate paths using Yen’s k -shortest path routing algorithm. The proposed algorithm runs Yen’s algorithm twice—from start to goal (stored in list A ) and from goal to start (list B ). This is a well-known technique to cover all possible h -signatures (Werner and Feld, 2014) (lines 4–5). As explained in Section 2.1, the positivity and negativity of h -signature depends on whether the path progresses from left to right or right to left. The algorithm thereby counts the number of paths in A (assigned to $N_{A}$ ), while concatenating two list A and B as P (lines 6–7).

The next step is to determine the shortest path for each h -signature. For all paths found by Yen’s algorithm, our algorithm computes the h -signature in relation to the obstacles in the environment (line 9). It should be noted again that the sign of h -signature must be reversed if the path is from $x_{\text{goal}}$ to $x_{\text{start}}$ (lines 10–12). The algorithm will store the pair of the h -signature and shortest path with it in a dictionary D . If the computed h -signature of the new path γ (h(γ)) already exists in the dictionary, the path corresponding to that key is retrieved as $\gamma_{\text{tmp}}$ and compared with the length of the new path γ . If the new path γ is shorter than the already-stored path $\gamma_{\text{tmp}}$ , the algorithm shorten the new path γ using line-of-sight algorithm (Yang, 2011) and updates the value of the dictionary with the new path (lines 13–20). If the computed h -signature h(γ) does not exist in dictionary D , the algorithm shortens the path γ and update the dictionary with the new key h(γ) and the value γ (lines 22–23).

In the k -shortest path routing algorithm, the value of k is typically determined empirically because the value depends on the map size (resolution) and number of obstacles. Here, we set the value of k to 120.

Algorithm 1 Enumeration of possible h -signatures.

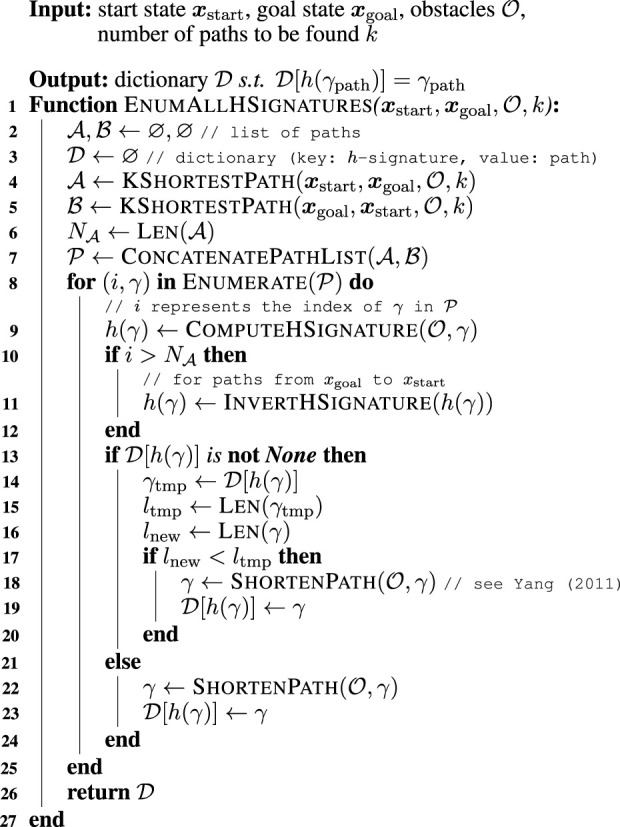

Algorithm 2 Computation of Reference Path.

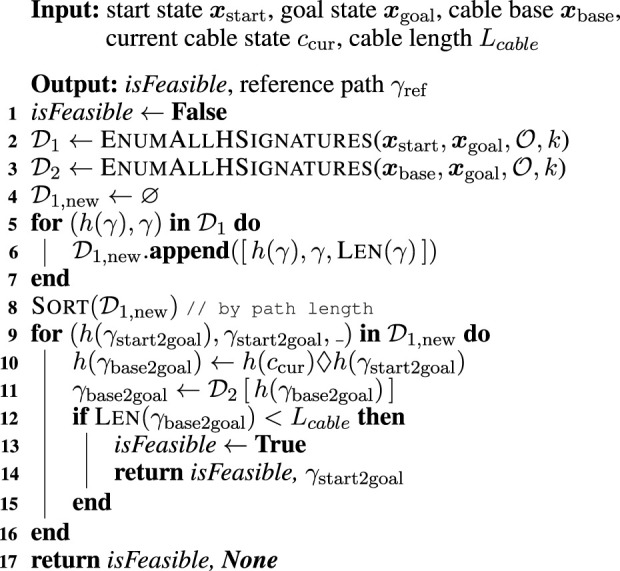

### 4.2 Step two—Computing reference path

Algorithm 1 allows us to find the possible kinds of h -signatures in a given environment and the shortest paths with each h -signature. Algorithm 2 then determines which of these h -signatures is the best by considering the placement and length of the cable. The path found by Algorithm 2 is used as a reference path in DRL to improve the efficiency of the agent’s path search. Here we describe the procedures for finding the best path as a reference path under cable constraints.

**FIGURE 3:**
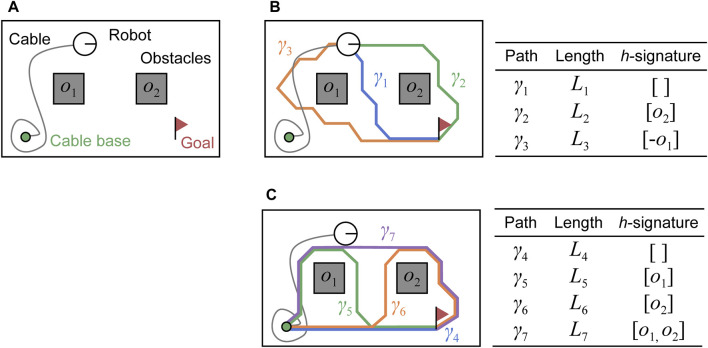
Example of paths stored in two dictionaries $D_{1}$ and $D_{2}$ in Algorithm 2. **(A)** Initial configuration of the tethered mobile robot and environment with two obstacles. **(B)** Paths connecting $x_{\text{start}}$ and $x_{\text{goal}}$ in the environment. They are distinct in terms of homotopy class, shortest in each class, and stored into a dictionary $D_{1}$ in the algorithm. The three paths are denoted as $\gamma_{1},\gamma_{2}$ , and $\gamma_{3}$ and their lengths are $L_{1},L_{2}$ , and $L_{3}$ , respectively. **(C)** Paths connecting $x_{\text{base}}$ and $x_{\text{goal}}$ . In this case, there exists four different homotopy classes. They are expressed as $\gamma_{4},\gamma_{5},\gamma_{6}$ , and $\gamma_{7}$ with lengths $L_{4},L_{5},L_{6}$ , and $L_{7}$ , respectively.

Algorithm 2 first initializes a Boolean flag with false that checks for the existence of a reachable path to the target position with consideration of cable placement and length (line 1). When this flag is returned as false, it means that the goal is too far away for the length of the cable. This algorithm then enumerates the h -signatures for two different configurations: paths from $x_{\text{start}}$ to $x_{\text{goal}}$ (stored in dictionary $D_{1}$ ) and from $x_{\text{base}}$ to $x_{\text{goal}}$ (dictionary $D_{2}$ ) (lines 4–5). An example of these two sets of paths is visualized in Figure 3. The paths connecting $x_{\text{start}}$ and $x_{\text{goal}}$ are classified into three types of homotopy classes. The visualized paths are the shortest in each of these classes. Let $\gamma_{1},\gamma_{2}$ , and $\gamma_{3}$ be the three shortest paths. The paths have their lengths $L_{1},L_{2}$ , and $L_{3}$ , respectively, and they are assumed to satisfy $L_{1}<L_{2}<L_{3}$ . For the paths connecting $x_{\text{base}}$ and $x_{\text{goal}}$ , four different homotopy classes are found. Let $\gamma_{4},\gamma_{5},\gamma_{6}$ , and $\gamma_{7}$ denote the four paths in the figure and their lengths $L_{4},L_{5},L_{6}$ , and $L_{7}$ , respectively. For simplicity of explanation, we assume that $L_{4}<L_{\text{cable}}<L_{5}<L_{6}<L_{7}$ (where $L_{\text{cable}}$ is the cable length).

The next step is to find the shortest path in the dictionary $D_{1}$ that is unrestricted by cable placement and length. The algorithm first creates an empty list $D_{1,new}$ and calculates the path length for all paths in $D_{1}$ (lines 4–7) and then sorts the list $D_{1,new}$ by path length from shorter to longer (line 8). This sorting allows us to check the cable constraints from shorter paths in $D_{1,new}$ in the subsequent step.

After sorting the list $D_{1,new}$ the algorithm retrieves a set of elements—a candidate path $\gamma_{\text{start}2\text{goal}}$ and its h -signature $h\left(\gamma_{\text{start}2\text{goal}}\right)$ — from the list one by one (line 9). Here we denote the current cable placement as $c_{\text{cur}}$ . By concatenating two h -signatures $h\left(c_{\text{cable}}\right)$ and $h\left(\gamma_{\text{start}2\text{goal}}\right)$ , the h -signature of a path connecting $x_{\text{base}}$ and $x_{\text{goal}}$ can be obtained (line 10). The proposed algorithm then gets the shortest path with its h -signature that connects $x_{\text{base}}$ and $x_{\text{goal}}$ by referring to the dictionary $D_{2}$ ; this is expressed as $\gamma_{\text{base}2\text{goal}}$ (line 11). Now imagine the following situation: when the robot tracks this path precisely, the cable becomes taut; therefore, we can say that the path $\gamma_{\text{base}2\text{goal}}$ is feasible if its length is less than the cable length $L_{\text{cable}}$ (lines 12–15).

We explain the process in lines 9–15 using the visualized example in Figure 3. In line 9, we first retrieve the elements with shortest path from $D_{1,new}$ , which are with regard to path $\gamma_{1}$ , because we assume $L_{1}<L_{2}<L_{3}$ . We can then obtain the h -signature of the final cable placement when the robot tracks the path $\gamma_{1}$ by computing $h\left(\gamma_{\text{base}2\text{goal}}\right)=h\left(c_{\text{cur}}\right)♢h\left(\gamma_{1}\right)=\left[o_{1}\right]♢\left[\;\right]=\left[o_{1}\right]$ (line 10). This h -signature corresponds to the one of path $\gamma_{5}$ ; thus, we can find the minimum cable length to achieve the goal by tracking path $\gamma_{1}$ (line 11). Given the aforementioned assumption $L_{4}<L_{\text{cable}}<L_{5}<L_{6}<L_{7}$ , we can determine that this path $\gamma_{1}$ cannot lead the robot to the goal with this cable placement and length constraint (lines 12–15). We extract the next element from $D_{1,new}$ — path $L_{2}$ . The h -signature of the final cable placement can be computed as $h\left(\gamma_{\text{base}2\text{goal}}\right)=h\left(c_{\text{cur}}\right)♢h\left(\gamma_{2}\right)=\left[o_{1}\right]♢\left[o_{2}\right]=\left[o_{1},o_{2}\right]$ . As shown in Figure 3, this h -signature is the same as the one for path $\gamma_{7}$ . The length of this path exceeds the maximum cable length, and we can say that the path $\gamma_{2}$ is infeasible under the cable constraints. Finally, we extract the elements with path $\gamma_{3}$ from $D_{1,new}$ . The h -signature of final cable placement can be calculated by $h\left(\gamma_{\text{base}2\text{goal}}\right)=h\left(c_{\text{cur}}\right)♢h\left(\gamma_{1}\right)=\left[o_{1}\right]♢\left[-o_{1}\right]=\left[\;\right]=h\left(\gamma_{4}\right)$ . Considering $L_{4}<L_{\text{cable}}$ , we can conclude that the path $\gamma_{3}$ is feasible.

If all paths in the list $D_{1,new}$ are turned out to be infeasible, the assigned goal cannot be reachable from the current robot’s configuration. In actual implementation, we recommend that this algorithm displays a warning to the user: Please redefine your goal because it is unreachable.

### 4.3 Step three—training agent

The reward function includes two terms: the pure RL term, $f\left(s_{t},a_{t}\right)$ , and the reference path term, $g\left(s_{t},a_{t},\gamma_{\text{ref}}\right)$ , as shown in Equation 1.

The reward function, $f\left(s_{t},a_{t}\right)$ , which awards penalties for any contact between the robot, cable, and obstacles, is expressed as

$$f\left(s_{t},a_{t}\right)=\left\{\begin{array}{cc} r_{\text{goal}}\; & \text{if}\;\text{the}\;\text{robot}\;\text{reaches}\;\text{the}\;\text{goal,}\\ p_{robot\_obstacle}\; & \text{if}\;\text{the}\;\text{robot}\;\text{collides}\;\text{with}\;\text{an}\;\text{obstacle}\\ p_{cable\_obstacle}\,\left(=p_{\text{co}}\right)\; & \text{if}\;\text{Equation}\;8\;\text{holds,}\\ p_{cable\_robot}\,\left(=p_{\text{cr}}\right)\; & \text{if}\;\text{Equation}\;9\;\text{holds,}\\ p_{\text{rotation}}\; & \text{when}\;\text{the}\;\text{robot}\;\text{rotates,}\\ p_{\text{time}}\; & \text{at}\;\text{every}\;\text{time}\;\text{steps.} \end{array}\right)$$ (2)

The reward $r_{\text{goal}}$ is given for arriving at the goal. The penalties $p_{robot\_obstacle}$ , $p_{cable\_obstacle}$ , and $p_{cable\_robot}$ are given at the time each contact event occurs. The penalty $p_{\text{rotation}}$ suppresses the robot’s *in situ* turn because remaining in a safe place is not the intended policy. The penalty $p_{\text{time}}$ is given at each time step to encourage the robot to move toward the goal in fewer steps.

The exact value of the positive/negative rewards was determined through experiments. In this study, we set $r_{\text{goal}}=50$ , $p_{\mathrm{robot\_obstacle}}=-1.0$ , $p_{\mathrm{cable\_obstacle}}=-1.0$ , $p_{\mathrm{cable\_robot}}=-0.5$ , $p_{\text{rotation}}=-0.1$ , and $p_{\text{time}}=-0.001$ . The reward $r_{\text{goal}}$ is 50 times larger than $p_{cable\_obstacle}$ in absolute terms. The magnitude of $r_{\text{goal}}$ could depend on the map size and the density of obstacles of a target problem. In this reward design, for a 25-s simulation with 250 timesteps, up to 50 cable-obstacle contacts can be covered by the reward. And in our evaluation, the number of contacts was kept below that level on average (see Table 2); hence, this reward design was appropriate to this map size and obstacle density. It should be noted that the penalty $p_{\text{time}}$ is quite small but necessary for keeping the robot move toward the goal. The validity of this reward design will be examined through sensitivity study in Section 6.3.3.

The reward function $g\left(s_{t},a_{t},\gamma_{\text{ref}}\right)$ with respect to the reference path imposes a penalty for deviating from the path and a reward for progressing along the path. This is written as Equation 3:

$$g\left(s_{t},a_{t},\gamma_{\text{ref}}\right)=\left\{\begin{array}{cc} r_{\text{progress}}\; & \text{for reaching each waypoint,}\\ p_{\text{deviation}}\; & \text{for exceeding threshold.} \end{array}\right.$$ (3)

Let $d_{\text{nearest}}$ be the distance from the current robot position to the nearest waypoint on the reference path and $r_{\text{rbt}}$ be the radius of the robot. Then, penalty $p_{\text{deviation}}$ can be expressed as Equation 4:

$$p_{\text{deviation}}=\left\{\begin{array}{cc} d_{\text{nearest}}-d_{\text{th}}\; & \text{if }d_{\text{nearest}}>d_{\text{th}}\text{,}\\ 0\; & \text{otherwise,} \end{array}\right.$$ (4)

where $d_{\text{th}}$ is the threshold for determining the deviation from the reference path, and we set $d_{\text{th}}=2.5\cdot r_{\text{rbt}}$ . In this study, we set $r_{\text{progress}}=r_{\text{goal}}/n_{\gamma_{\text{ref}}}$ to reach each waypoint, where $n_{\gamma_{\text{ref}}}$ is the number of nodes in $\gamma_{\text{ref}}$ . The ablation study of the reference path will be examined in Section 6.3.4.

## 5 Simulation model

We present a tethered robot model that uses a kinematics-based robot with a position-based cable. Although this model lacks mechanical fidelity, it offers computational efficiency, which is useful for iterative RL simulations.

### 5.1 Kinematics-based robot model

**FIGURE 4:**
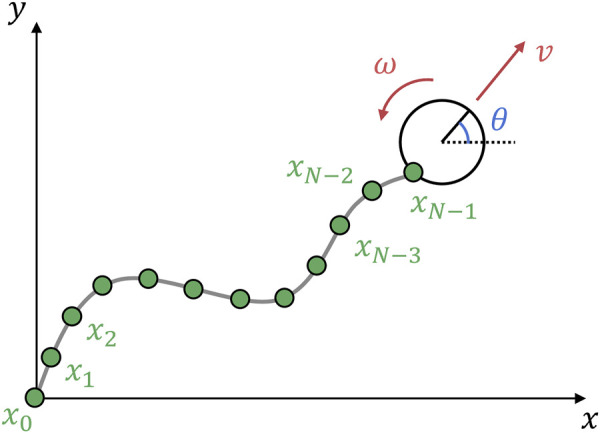
Tethered mobile robot model used in this study: kinematics-based robot model and position-based cable model. The one end of the cable $x_{0}$ is attached to the ground (origin of the coordinates) and $x_{N-1}$ is the rear side of the robot, where N is the number of nodes. The position of $x_{N-1}$ is updated in response to the robot’s straight and rotational motion, and it propagates to the other cable nodes.

In this study, we used the velocity motion model (Thrun, 2002) (Figure 4). The command velocity can be input directly into the model. The robot state is defined as $x={\left[\;x,\;y,\;\theta \;\right]}^{⊤}$ , and the control input to the robot as $u_{t}={\left[\;v_{t},\omega_{t}\;\right]}^{⊤}$ , where $v_{t}$ is the translational velocity and $\omega_{t}$ is the angular velocity at discrete time t . The kinematics of this robot is simply written as Equation 5:

$$\left[\begin{array}{c} x_{t+1}\\ y_{t+1}\\ \theta_{t+1} \end{array}\right]=\left[\begin{array}{c} x_{t}\\ y_{t}\\ \theta_{t} \end{array}\right]+\left[\begin{array}{c} v_{t}\cos\theta_{t}\\ v_{t}\sin\theta_{t}\\ \omega_{t} \end{array}\right]\Delta t.$$ (5)

### 5.2 Position-based cable model

A cable is modeled as a chain of nodes; when one cable node is pulled, the rest of the nodes follow. This method is called a geometry- or position-based model and is often used in computer graphics. We extended the model in Brown et al. (2004) for a tethered mobile robot to update the cable nodes from two end nodes: 1) the propagation of the robot motion to the cable, and 2) the application of the fixed node constraint to the entire cable nodes. On the first side, from the robot motion to the cable nodes, the node positions are updated as follows:

$$\begin{array}{ll} x_{i,\;new}=x_{i+1,\;new} & \;+\frac{\left(x_{i,\;old}-x_{i+1,\;new}\right)}{\left|x_{i,\;old}-x_{i+1,\;new}\right|}\\ & \left(i=N-2,N-3,\ldots ,0\right). \end{array}$$ (6)

For the second side, the node positions are updated using

$$\begin{array}{ll} x_{i,\;new}=x_{i-1,\;new} & \;+\frac{\left(x_{i,\;old}-x_{i-1,\;new}\right)}{\left|x_{i,\;old}-x_{i-1,\;new}\right|}\\ & \left(i=1,2,\ldots ,N-1\right). \end{array}$$ (7)

Equation 7 updates in the opposite direction to Equation 6 and propagates zero displacement because the endpoint $x_{0}$ is fixed, to the remaining cable nodes.

### 5.3 Contact detection

To detect cable-obstacle contact, we use the Liang-Barsky algorithm (Liang and Barsky, 1984), which is a line clipping algorithm. Although this algorithm originally identifies the overlaps between a rectangle and a line segment, it can also be used for contact detection. The line segment with the end points $x_{0}$ and $x_{1}$ is expressed in parametric form as $x\left(t\right)=x_{0}+\alpha \left(x_{1}-x_{0}\right)$ , using a *clipping parameter* α . Here we determine the four clipping parameters α where the line intersects the four edges of the rectangle. We can determine the maximum, $\alpha_{\max}$ , and minimum, $\alpha_{\min}$ , of these parameters. The line segment and rectangle are said to intersect if the following Equation 8 holds:

$$0\leq \alpha_{\min}\leq \alpha_{\max}\leq 1.$$ (8)

To detect cable-robot contact, we compute the distance between the center of the robot, $x_{\mathrm{rbt}}$ , and the cable nodes, $x_{i}$ , as Equation 9:

$$\left\|x_{\text{rbt}}-x_{i}\right\|\leq r_{\text{rbt}}\;\left(i=0,1,\ldots ,N-2\right),$$ (9)

where $r_{\text{rbt}}$ denotes the robot’s radius (set to 0.5). It should be noted that cable node $x_{N-1}$ is excluded from the above equation because it represents the robot-cable connection point.

## 6 Simulation results and discussion

This section evaluates the proposed method in the two distinct scenarios explained in Section 3: **unreeling the cable** (Scenario 1) and **handling the deployed cable** (Scenario 2). All simulations were executed on an Intel(R) Core(TM) i7-12700 CPU clocked at 2.10 GHz, NVIDIA GeForce RTX 3070, Python 3.8, and PyTorch 1.13. A single learning with 300,000 total timesteps, as defined in stable-baselines3 (Raffin et al., 2021), required approximately 8 min.

### 6.1 RL environment

We formulate the TCFPP as an MDP expressed by the tuple: (S,A,T,R) , as explained in Section 2-B. To manage the computational cost, the state space S , which is represented as $x={\left[\;x,y,\theta \;\right]}^{⊤}$ is discretized with specified intervals: 0.2 for (x,y) and π/18 for θ . The action space, A , which is expressed as a pair of linear and angular velocities, (v,ω) , contains three minimal actions: turn right (0.0,−2.0) , go straight (1.0,0.0) , turn left (0.0,2.0) . The state transition function T is deterministic. It should be noted here that the proposed method examined in this study does not need to consider the explicit expression of geometric or kinematic physical quantities: the unit of length can be arbitrarily defined, while the unit of time is seconds.

The Deep Q-Network used in this study comprises the main and target networks with a three-layer neural network structure. The state of the robot is processed in the input layer, followed by two middle layers of 256 units each with ReLU activation, and finally, an output layer that maps to the robot’s actions. The termination condition for an episode is two-fold: reaching the goal and colliding with an obstacle. The agent receives a positive reward, $r_{\text{goal}}$ , in the first case and a negative reward, $p_{robotobstacle}$ , in the second case. The simulation parameters and hyperparameters used in our method are listed in Table 1.

**TABLE 1:** Hyperparameters in deep Q-Network.

Parameter	Value
Optimizer	Adam (Kingma and Ba, 2014)
Learning rate	0.001
Discount factor	0.9
Buffer size	10,000
Mini-batch size	32
Total episodes	300,000
Target update interval	30
Learning starts	5,000
Exploration fraction	0.6
Initial exploration rate	0.9
Final exploration rate	0.05

### 6.2 Experimental setup

We tested our algorithm in three environments: **Dots**, **Two bars**, and **Complex** environments (Figure 6). These datasets were inspired by Bhardwaj et al. (2017) and serve different purposes:

• The **Dots** maps represent cluttered environments in which an agent must consider multiple path patterns in the sense of homotopy classes.
• The **Two bars** maps represent simplified indoor environments with two rooms separated by a narrow passage, in which the agent can take multiple path patterns in the sense of geometry (not homotopy). The focus is on how distant from the obstacles the agent chooses a path.
• The **Complex** maps represent unstructured indoor environments with various numbers and shapes of obstacles. In these environments, the agent must consider both homotopy and geometry, which makes the path-finding task complex.

In this study, we manually defined five environments for each type and validated our proposed method in 20 cases by considering the initial cable configuration.

The generation of initial cable placements presents unique challenges for Scenario 2 because the obstacles must be considered. In this study, we generated a reasonable initial cable configuration by following a sequential process: 1) run a simulation in the unreeling cable scenario (Scenario 1); 2) record the final robot and cable placements; 3) retrieve the recorded placements and set them as the initial setting in the handling deployed cable scenario (Scenario 2); 4) assign the next goal position and start the next simulation; 5) repeat ii) to 6) until the completion of the specified number of simulations.

To evaluate contact avoidance, we used the following metrics: path length $L_{\text{path}}$ , number of cable-robot contacts $N_{cr}$ , and number of cable-obstacle contacts $N_{co}$ . The path length is defined as the sum of the distances between the adjacent waypoints. This metric is considered because the contact avoidance actions can increase the overall path length. The two contacts were counted at each discrete simulation timestep (0.1 s) in the path tracking simulation using the pure pursuit algorithm (Coulter, 1992) and normalized by converting them into times per minute. This normalization allows for a fair evaluation that considers the differences in the total simulation time. A smaller value of these metrics indicates a better performance of the algorithm.

### 6.3 Results

We compared our proposed method (TCFPP) with two classical path planning methods augmented with h -signature information as baselines. The two methods are Dijkstra’s algorithm (Dijkstra, 1959) and Voronoi roadmap (Lozano-Pérez and Wesley, 1979); they are referred to as Homotopic Dijkstra’s algorithm and Homotopic Voronoi roadmap in this study. We used Dijkstra’s algorithm for its path shortness and the Voronoi roadmap for its effectiveness in maintaining the distance from obstacles.

The reason we provide h -signature information to the baselines is for fair comparison. Thus, the two classical and homotopic methods searched for a path that had the same h -signature as the path found by TCFPP. This Homotopic Dijkstra’s algorithm is essentially the same as that of Kim et al. (2014), a state-of-the-art (SOTA) in this field, although it does not deal with cable-obstacle and cable-robot avoidance.

#### 6.3.1 Qualitative evaluation on path similarity

**FIGURE 5:**
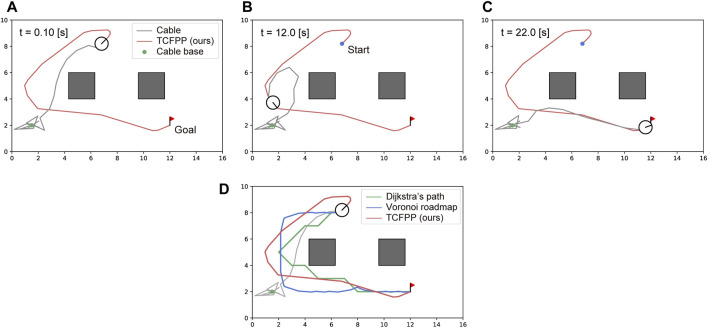
Typical simulation result. **(A–C)** The snapshots of path tracking generated by our method are shown. The circular robot, whose line segment represents its heading, is connected with the environment via a cable (gray line) at a point (green dot). The red line represents the path generated by our method, which connects the start point (blue dot) and goal point (red flag). **(D)** The visual comparison of the generated path by TCFPP with two classical planning methods is depicted.

**FIGURE 6:**
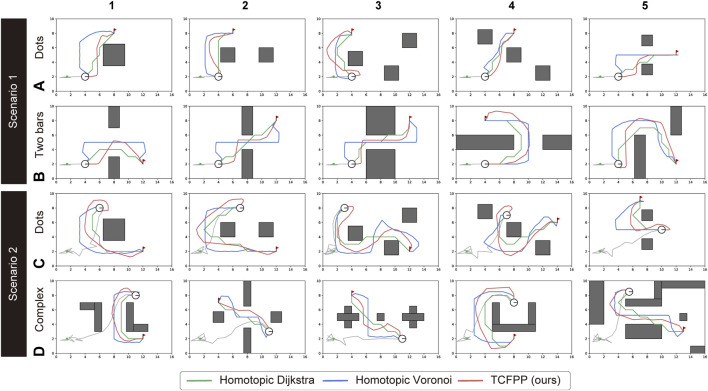
Path comparison for all 20 configurations (5 maps × 2 environment types × 2 scenarios). The two types of environments for Scenario 1 are **(A)** and **(B)**, and for Scenario 2, they are **(C)** and **(D)**. The three path planning algorithms shared the best homotopy class of the paths; however, they generated different paths with respect to the path shape, path length, and proximity to the obstacles. The green lines represent the Homotopic Dijkstra’s algorithm, the blue lines represent the Homotopic Voronoi roadmap, and the red lines represent the proposed method.

Figure 5 shows snapshots of a typical path tracking simulation and the path similarity with the baselines. Figures 5A–C show the sequential movement of the robot that follows the path obtained by TCFPP. Figure 5D shows the paths of the three methods with the same h -signature. All the simulations performed in this study are depicted in Figure 6. From this figure, it can be seen that our method shows greater expansion at waypoints with higher curvature than Homotopic Dijkstra’s method, which reduces the excessive movement for cable-obstacle avoidance seen in Homotopic Voronoi roadmap, particularly when the robot unreels the cable (Figures 6A, B). When the robot handles the deployed cable (Figures 6C, D), our method becomes more conservative in balancing both cable-robot and cable-obstacle contacts.

#### 6.3.2 Quantitative evaluation on contact avoidance

Table 2 presents a comparison of the quantitative results of the three metrics: path length $L_{\text{path}}$ , number of cable-obstacle contacts $N_{co}$ , and number of cable-robot contacts $N_{cr}$ . The results are the averages of five simulations. Regarding the path length, for all cases, the proposed method generated paths that were 20.4% longer than those generated by Homotopic Dijkstra’s algorithm and 12.4% shorter than those generated by the Homotopic Voronoi roadmap. In the two cases of Scenario 1, no cable-robot contact was detected, except for one simulation of Homotopic Voronoi roadmap. The exceptional result was that the Voronoi path passed close to the cable base, where the cable was located before it was pulled out. This is because we provided the h -signature of the path to Homotopic Voronoi planner, but not information about the cable placement. For cable-obstacle avoidance, the proposed method outperformed Homotopic Dijkstra’s algorithm but underperformed Homotopic Voronoi roadmap.

**TABLE 2:** Quantitative results over five simulations in each case. The values are the averages of five simulations (best in bold). The metrics $L_{\text{path}}$ , $N_{\text{co}}$ , and $N_{\text{cr}}$ denote the path length, the number of cable-obstacle contacts, and the number of cable-robot contacts, respectively. The latter two were counted for each time step, 0.1 s, and normalized by the total simulation time as 1 min.

	Scenario 1 (Dots)			Scenario 1 (Two bars)			Scenario 2 (Dots)			Scenario 2 (Complex)		
	$L_{\text{path}}$	$N_{\text{co}}$	$N_{\text{cr}}$	$L_{\text{path}}$	$N_{\text{co}}$	$N_{\text{cr}}$	$L_{\text{path}}$	$N_{\text{co}}$	$N_{\text{cr}}$	$L_{\text{path}}$	$N_{\text{co}}$	$N_{\text{cr}}$
Homotopic Dijkstra	**7.93**	8.37	**0.00**	**12.0**	15.1	**0.00**	**13.2**	21.1	28.4	**11.1**	12.4	40.6
Homotopic Voronoi	10.64	**1.13**	38.5	16.8	**0.00**	**0.00**	18.7	**0.00**	40.8	14.8	**1.52**	62.7
TCFPP (ours)	8.92	1.20	**0.00**	14.3	10.6	**0.00**	17.3	0.55	**17.6**	13.2	1.60	**37.3**

#### 6.3.3 Sensitivity study on penalty for cable-robot contact

**FIGURE 7:**
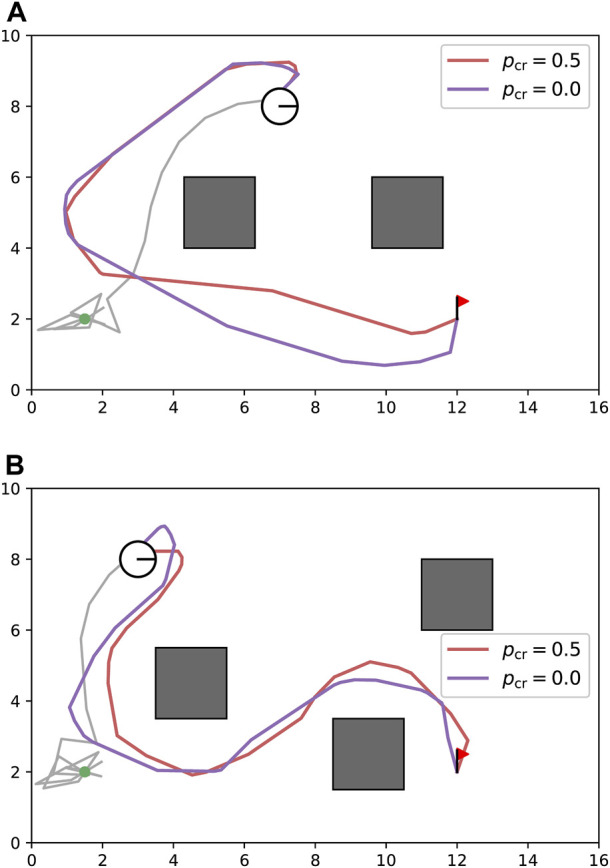
Sensitivity study on cable-robot penalty $p_{\text{cr}}=0.0$ . The decrease in $p_{\text{cr}}$ resulted in the planned paths having on average larger distances between the obstacles and the robot. **(A)** Path comparison in an example case of Figure 6 C2. **(B)** Path comparison in an example case of Figure 6 C3.

The proposed reward design was not based on physical parameters; therefore, it was difficult to understand intuitively whether the values and weights of the rewards were appropriate. Therefore, we evaluated the impact of the penalties, $p_{\text{co}}$ and $p_{\text{cr}}$ , on the performance by changing the value of $p_{\text{cr}}$ in Equation 2, while keeping $p_{\text{co}}$ constant. We ran five additional simulations for Scenario 2, with $p_{\text{cr}}$ set to 0.0 and 1.0. The results in Table 3 reveal variations in the agent behavior. With $p_{\text{cr}}=0.0$ , the agent only considers cable-obstacle avoidance, thereby resulting in the generation of more distant paths from the obstacles in sufficiently large areas to avoid motion (Figure 7). From Table 3, it can be observed that the number of cable-robot contacts significantly increased; and the number of cable-obstacle contacts was less variable than that in the cases of $p_{\text{cr}}=0.5$ .

**TABLE 3:** Sensitivity study on the penalty value for cable-robot contact. This penalty can adjust whether the agent can or cannot step over the cable. When $p_{\text{cr}}=0$ , the robot was deadlocked by the cable and could not reach the goal in some cases.

$p_{\text{cr}}$	Scenario 2 (Dots)			
	Success Rate	$L_{\text{path}}$	$N_{\text{co}}$	$N_{\text{cr}}$
1.0	3/5	15.6^a^	0.00^a^	6.22^a^
0.5	5/5	17.3	0.55	17.6
0.0	5/5	18.0	1.04	44.10

^a^ Average of three successful results.

**FIGURE 8:**
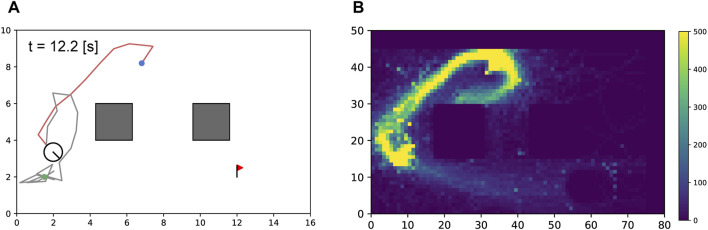
Sensitivity study on cable-robot penalty $p_{\text{cr}}=1.0$ . **(A)** The robot blocked by the cable. The cable was seen as an obstacle that could not be stepped over because $p_{\text{co}}=p_{\text{cr}}=1.0$ holds; thus, the robot was deadlocked by the cable. **(B)** Heatmap visualizing the distribution of agent visits to each cell in the map over 300,000 training steps. In this case, the agent did not acquire the desired policy because it did not gain much experience in reaching the goal.

For $p_{\text{cr}}=1.0$ , the agent regards the cable as an obstacle that the robot can step over because the equation $p_{\text{co}}=p_{\text{cr}}$ holds true, which potentially causes path-finding issues. In some cases (two out of five simulations), the agent could not find a path to the goal because of the deadlock imposed by the cable (Figure 8A). The heatmap in Figure 8B shows the number of visits during the training process for each discretized cell, which shows that the search area could not be expanded sufficiently owing to blockage by the cable. From Table 3, both $N_{cr}$ and $N_{cr}$ reduced compared with the cases where $p_{\text{cr}}=0.5$ ; however, the success rate also decreases. This analysis suggests that a reward design with $p_{\text{cr}}=0.5$ is more effective at minimizing both types of contacts as well as the deadlock.

#### 6.3.4 Ablation study on reference path

**FIGURE 9:**
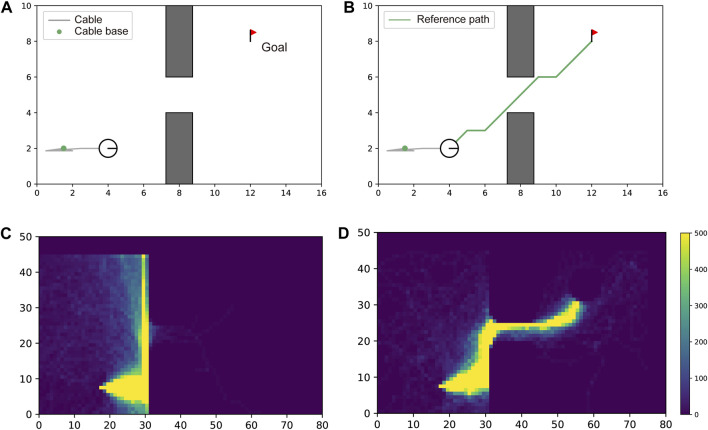
Effectiveness of reference path for learning efficiency. In **(A, C)**, the robot could not traverse the gap without guidance of a reference path. The number of times the agent visited each cell of the map during the training process is visualized as a heatmap. This heatmap shows the lack of experience of the agent in reaching the goal. In **(B, D)**, the robot successfully traversed the gap with guidance from the reference path (green line). The heatmap shows the efficient search along the path.

The proposed reward design has two terms: a pure RL term and a reference path term, as presented in Equation 1. We evaluated the effectiveness of providing information about the reference path to the agent. When we do not use the reference path information, we simply eliminate the second term of the reward function $g\left(s_{t},a_{t},\gamma_{\text{ref}}\right)$ . Figure 9 shows the difference of the exploration area with and without the reference path in RL. A comparison of the two heatmaps reveals that without the reference path, the agent could not find a path that traversed between gaps in 300,000 timesteps. However, with the reference path, the agent efficiently explored the state space and successfully found the optimal path, free from contacts.

#### 6.3.5 Limitations and possible extensions

Although the proposed method demonstrated successful improvements in cable-robot and cable-obstacle avoidance in a specific environment, a versatile policy was not obtained. A possible extension would be to use curriculum learning (Soviany et al., 2022), where the learning environment gradually becomes more difficult as the training progresses. The key challenge is expected to be the formulation of navigational difficulties for a tethered mobile robot. To this end, considering realistic parameters of specific hardware and work environments would help. The parameters are, for example, the wheel diameter of the robot and cable thickness, which are crucial for accurately modeling cable overstepping; additionally considering cable-obstacle and cable-ground friction to more realistically simulate cable and obstacle interactions, *e.g.*, displacement of lightweight obstacles by the cable.

## 7 Conclusion

This paper presented TCFPP, a tangle- and contact-free path planning for a tethered mobile robot that minimizes the cable-obstacle and cable-robot interactions with cable length constraints. Path planning for a tethered mobile robot is a challenging task due to the underactuated nature of the cable and due to the interactions between the cable, the robot, and the obstacles that potentially endanger the safe operation of the robot. We formulated this task as a reinforcement learning problem and trained the agent in simulation with Deep Q-Network. Our reward function consisted of two main parts: the first was to give penalties against cable-obstacle and cable-robot contacts, and the second was to give a penalty against the deviation from the precomputed reference path and to give a reward for the motion along the path. This reward design enabled finding paths that minimize the cable-obstacle and cable-robot contacts while efficiently searching in the vicinity of the reference path. The proposed method was tested in the two distinct scenarios—unreeling cable and handling deployed cable. Compared with classical path planning algorithms, the proposed method generated a path that balances its shortness with the avoidance of cable-obstacle and cable-robot contacts.

Future work will focus on incorporating curriculum learning to enhance the adaptability of the policy to various environments and on integrating realistic parameters such as wheel diameter and cable thickness. These factors can enable the robot to acquire a versatile policy, and further enhance the safety of tethered robot deployment in real-world scenarios.

## Data Availability

The raw data supporting the conclusions of this article will be made available by the authors, without undue reservation.

## References

[B1] Abad-Manterola P. Nesnas I. A. Burdick J. W. (2011). “Motion planning on steep terrain for the tethered axel rover,” in *2011 IEEE international Conference on Robotics and automation* (IEEE), 4188–4195.

[B2] Bhardwaj M. Choudhury S. Scherer S. (2017). Learning heuristic search via imitation. arXiv. arXiv:1707.03034 [preprint].

[B3] Brass P. Vigan I. Xu N. (2015). Shortest path planning for a tethered robot. Comput. Geom. 48, 732–742. 10.1016/j.comgeo.2015.06.004

[B4] Brown J. Latombe J.-C. Montgomery K. (2004). Real-time knot-tying simulation. Vis. Comput. 20, 165–179. 10.1007/s00371-003-0226-y

[B5] Coulter R. C. (1992). Implementation of the pure pursuit path tracking algorithm. Tech. Rep. Carnegie-Mellon UNIV Pittsburgh PA Robotics INST.

[B6] Dijkstra E. (1959). A note on two problems in connexion with graphs. Numer. Math. 1, 269–271. 10.1007/bf01386390

[B7] Igarashi T. Stilman M. (2010). “Homotopic path planning on manifolds for cabled mobile robots,” in Algorithmic foundations of robotics IX (Springer), 1–18.

[B8] Kim S. Bhattacharya S. Kumar V. (2014). “Path planning for a tethered mobile robot,” in 2014 IEEE international Conference on Robotics and automation (IEEE), 1132–1139.

[B9] Kim S. Likhachev M. (2015). “Path planning for a tethered robot using multi-heuristic a with topology-based heuristics,” in *2015 IEEE/RSJ international Conference on intelligent Robots and systems* (IEEE), 4656–4663.

[B10] Kingma D. P. Ba J. (2014). Adam: a method for stochastic optimization. *arXiv preprint arXiv:1412.6980* .

[B11] Liang Y.-D. Barsky B. A. (1984). A new concept and method for line clipping. ACM Trans. Graph. 3, 1–22. 10.1145/357332.357333

[B12] Lozano-Pérez T. Wesley M. A. (1979). An algorithm for planning collision-free paths among polyhedral obstacles. Commun. ACM 22, 560–570. 10.1145/359156.359164

[B13] Martz J. Al-Sabban W. Smith R. N. (2020). Survey of unmanned subterranean exploration, navigation, and localisation. IET Cyber-Systems Robotics 2, 1–13. 10.1049/iet-csr.2019.0043

[B14] Mnih V. Kavukcuoglu K. Silver D. Rusu A. A. Veness J. Bellemare M. G. (2015). Human-level control through deep reinforcement learning. nature 518, 529–533. 10.1038/nature14236 25719670

[B15] Nagatani K. Kiribayashi S. Okada Y. Otake K. Yoshida K. Tadokoro S. (2013). Emergency response to the nuclear accident at the fukushima daiichi nuclear power plants using mobile rescue robots. J. Field Robotics 30, 44–63. 10.1002/rob.21439

[B16] Ota K. Jha D. K. Oiki T. Miura M. Nammoto T. Nikovski D. (2020). Trajectory optimization for unknown constrained systems using reinforcement learning. arXiv Prepr. arXiv:1903.05751. 10.48550/arXiv.1903.05751

[B17] Raffin A. Hill A. Gleave A. Kanervisto A. Ernestus M. Dormann N. (2021). Stable-baselines3: reliable reinforcement learning implementations. J. Mach. Learn. Res. 22 (268), 1–8. 10.5555/3546258.3546526

[B18] Sahin A. Bhattacharya S. (2023). Topo-geometrically distinct path computation using neighborhood-augmented graph, and its application to path planning for a tethered robot in 3d. arXiv Prepr. arXiv:2306.01203. 10.48550/arXiv.2306.01203

[B19] Schreiber D. A. Richter F. Bilan A. Gavrilov P. V. Lam H. M. Price C. H. (2020). Arcsnake: an archimedes’ screw-propelled, reconfigurable serpentine robot for complex environments. IEEE International Conference on Robotics and Automation ICRA, 7029–7034.

[B20] Shapovalov D. Pereira G. A. (2020). “Exploration of unknown environments with a tethered mobile robot,” in *2020 IEEE/RSJ international Conference on intelligent Robots and systems* (IEEE), 6826–6831.

[B21] Shimada R. Ishigami G. (2023). “Path planning with cable-obstacles avoidance for a tethered mobile robot in unstructured environments,” in Proceedings of the 34th international symposium on space technology and science. 2023-k-04).

[B22] Soviany P. Ionescu R. T. Rota P. Sebe N. (2022). Curriculum learning: a survey. Int. J. Comput. Vis. 130, 1526–1565. 10.1007/s11263-022-01611-x

[B23] Thrun S. (2002). Probabilistic robotics. Commun. ACM 45, 52–57. 10.1145/504729.504754

[B24] Werner M. Feld S. (2014). “Homotopy and alternative routes in indoor navigation scenarios,” in *2014 international Conference on indoor Positioning and indoor navigation* (IEEE), 230–238.

[B25] Xavier P. G. (1999). “Shortest path planning for a tethered robot or an anchored cable,” in *Proceedings 1999 IEEE international Conference on Robotics and automation* (IEEE), 2, 1011–1017.

[B26] Yang K. (2011). Anytime synchronized-biased-greedy rapidly-exploring random tree path planning in two dimensional complex environments. Int. J. Control Automation Syst. 9, 750–758. 10.1007/s12555-011-0417-7

[B27] Yang T. Liu J. Wang Y. Xiong R. (2023). “Self-entanglement-free tethered path planning for non-particle differential-driven robot,” in *2023 IEEE international Conference on Robotics and automation* (IEEE), 7816–7822.

